# Expanding Window Compressed Sensing for Non-Uniform Compressible Signals

**DOI:** 10.3390/s121013034

**Published:** 2012-09-26

**Authors:** Yu Liu, Xuqi Zhu, Lin Zhang, Sung Ho Cho

**Affiliations:** 1 Key Lab of Universal Wireless Communications, Ministry of Education, Beijing University of Posts and Telecommunications, Beijing 100876, China; E-Mails: safiml@gmail.com (X.Z.); zhanglin@bupt.edu.cn (L.Z.); 2 Department of Electronics and Computer Engineering, Hanyang University, Seoul 133791, Korea; E-Mail: dragon@casp.hanyang.ac.kr

**Keywords:** compressed sensing, image compression, networked data, non-uniform compressible signal, random projection, unequal protection

## Abstract

Many practical compressible signals like image signals or the networked data in wireless sensor networks have non-uniform support distribution in their sparse representation domain. Utilizing this prior information, a novel compressed sensing (CS) scheme with unequal protection capability is proposed in this paper by introducing a windowing strategy called expanding window compressed sensing (EW-CS). According to the importance of different parts of the signal, the signal is divided into several nested subsets, *i.e.*, the expanding windows. Each window generates its own measurements using a random sensing matrix. The more significant elements are contained by more windows, so they are captured by more measurements. This design makes the EW-CS scheme have more convenient implementation and better overall recovery quality for non-uniform compressible signals than ordinary CS schemes. These advantages are theoretically analyzed and experimentally confirmed. Moreover, the EW-CS scheme is applied to the compressed acquisition of image signals and networked data where it also has superior performance than ordinary CS and the existing unequal protection CS schemes.

## 1. Introduction

Compressed sensing (CS) [1,2] is an emerging technology that promises low-complexity signal acquisition which is especially important for energy-constrained devices or large data sets, e.g., in high-resolution image signal processing, MRI imaging, *etc.* In the CS methodology, the signal which is sparse or compressible on a certain orthonormal basis can be successfully recovered from a highly incomplete set of samples, called measurements. Moreover, the measurements can be obtained by multiplying the signal by a randomly generated sensing matrix that is incoherent with most of the orthonormal basis. Thus, CS greatly improves the efficiency and decreases the complexity of signal compression.

**Figure 1.**
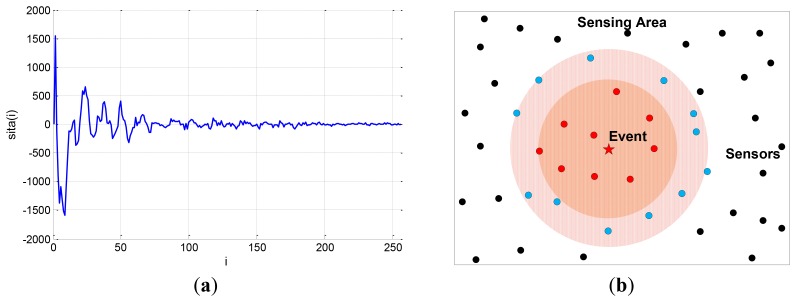
Examples of non-uniform compressible signals. (**a**) The DCT coefficients of the first 16 × 16 block in the first frame of “coastguard.qcif”. (**b**) The networked data scenario in wireless sensor networks.

The compressible signal can be indicated by the index of the sparse non-zero components and the value of the non-zero components, where the index of the sparse non-zero components is called sparse support. The ordinary CS algorithms are designed without any constraint on the distribution of the support of the sparse signal. Actually, many natural signals do have special support structures. For example, the image signal is compressible in the discrete cosine transform (DCT) basis or discrete wavelet transform (DWT) basis. The transform coefficients constitute a non-uniform compressible signal where the significant coefficients mostly appear at lower frequency, as shown in Figure 1(a). Another example is the CS of networked data [3] in wireless sensor networks, also called compressive wireless sensing (CWS) [4]. A large number of sensors are distributed in the sensing area, as shown in Figure 1(b), whose data are regarded as elements of a signal, called networked data. When an event occurs, the sensors around it will collect more significant data, so this networked data is also a kind of non-uniform compressible signal whose significant elements are placed within a certain part of the signal.

Considering these largely existing non-uniform compressible signals, if we can utilize the prior-knowledge of the support distribution to provide unequal protection for different parts of the signal, we can instinctively achieve better performance. In this paper, such a CS algorithm is proposed to improve the sensing and recovery efficiency for the non-uniform compressible signals, called the expanding window compressed sensing (EW-CS). The windowing method is inspired by the expanding window fountain codes [5,6], where the concept of expanding window is used in the binary field. We explore how to utilize the support window in a real field. With the prior knowledge that some parts of the signal are more important than others, the signal is divided into several nested subsets called windows. The more important the part is, and there will be more windows containing this part. Each window generates their own measurements with CS's random sensing matrix, so the more important part's elements are sampled in more measurements to guarantee higher protection level. This design makes the EW-CS scheme have more convenient implementation.

The proposed EW-CS scheme treats the different parts of the compressible signal discriminately to pursue the improved overall performance. The more attention on the more important parts is at the expense of the less important parts. Nevertheless, it is worthwhile to do this, because most of the energy or the useful information is contained in the more important parts. The overall performance improvement also has its prerequisite that is the prior knowledge of the rough non-uniform distribution of the significant elements. This prior information is not difficult to get in most cases. For image signals, the significant transform coefficients mostly appear at lower frequency. For networked data, the significant data is surrounding the emerging event. The above statements about the EW-CS are presented and proved from theoretical and numerical perspectives in our paper. Moreover, the EW-CS is applied to image signals and networked data, and shown to provide better performance than the ordinary CS and the existing unequal protection CS schemes.

The rest of this paper is organized as follows: in Section 2, the key issues of CS are briefly reviewed, and the related existing CS algorithms considering non-uniform compressible signals are presented and compared with our EW-CS algorithm. The details of the EW-CS scheme are introduced in Section 3. Section 4 is devoted to analyze the recovery error upper bounds of the proposed EW-CS to compare with the ordinary CS. In Section 5, the variation of basic EW-CS algorithm is discussed. The experimental results are shown and analyzed in Section 6. Section 7 concludes this paper and gives some directions for future work.

## 2. Related Work

### 2.1. Basics of Compressed Sensing

A signal *X* ∈ ℝ*^N^* is said to be sparse, if the transform coefficient vector Θ = { *θ_i_*, *i* = 1,2, …, *N*}, of *X* in an orthogonal basis Ψ*_N_*_×_*_N_* are mostly zero. The sparse support of Θ (or *X*) is defined as S = {*i: θ_i_* ≠ 0, *i* = 1,2,…, *N*}, and the length of *S* is the sparsity *K* = |*S*|, *K* ≪ *N*, so signal *X* is called as *K*-sparse signal. We consider a linear sparse case that *K* = *αN*, 0 < *α* ≪ 1 is a constant. If the most coefficients are not exactly zero but with small magnitude, signal *X* is called compressible signal. The elements with larger absolute value are regarded as significant. CS theory [1,2] states that *M* measurements of compressible signal *X* is enough for exact recovery where *M* < *N*. The sensing matrix Ф*_M_*_×_*_N_* should be incoherent with the representation basis Ψ*_N_*_×_*_N_* to guarantee the successful recovery. Fortunately, random matrices are largely incoherent with any fixed basis Ψ*_N_*_×_*_N_*. Thus, the measurements usually are called random projections when a random matrix is used in CS encoding. The CS encoding is quite simple that:

(1) $$Y=\Phi_{M\times N}X$$

where *Y* ∈ ℝ*^N^* is the measurement vector. The sensing rate is *R* = *M*/*N*. The reconstruction of CS is performed by solving the following constrained optimization problem:

(2) $$\begin{array}{cc} (\ell_{0})\min{\left\|\Theta \right\|}_{\ell_{0}} & s.t.Y=\Phi X=\Phi \Psi \Theta \end{array}$$

Although (*l*_0_) can give the unique solution with only *M* > 2*K* measurements, it is a NP hard problem. A relaxation of (*l*_0_) is the linear programming problem, also known as basis pursuit [7]:

(3) $$\begin{array}{cc} (\ell_{1})\min{\left\|\Theta \right\|}_{\ell_{1}} & s.t.Y=\Phi X=\Phi \Psi \Theta \end{array}$$

The restricted isometriy property (RIP) [8] is a sufficient condition for guarantee the equivalence of the (*l*_0_) and (*l*_1_) solutions with overwhelming probability. Let *δ_k_* be a smallest positive number such that:

(4) $$(1-\delta_{k}){\left\|X\right\|}_{\ell_{2}}^{2}\leq {\left\|\Phi X\right\|}_{\ell_{2}}^{2}\leq (1+\delta_{k}){\left\|X\right\|}_{\ell_{2}}^{2}$$

holds for all *k*-sparse signals, we say sensing matrix Ф satisfies the RIP with *δ_k_*. Random generated sensing matrixes satisfy RIP with high probability [9]. When the measurements are polluted by noise, *i.e.*, *Y* = Ф*X* + *e*, where *e* ∈ ℝ*^M^*, the (*l*_1_) can be further improved by solving the Lasso [10], or basis pursuit denoising [7]:

(5) $$\begin{array}{cc} (\text{Lasso})\min{\left\|\Theta \right\|}_{\ell_{1}} & s.t.{\left\|Y-\Phi X\right\|}_{\ell 2}\leq ɛ \end{array}$$

where *ε* bounds the amount of noise *e*. The (*Lasso*) is again a convex programming problem that can be solved with many classical algorithms, e.g., gradient projection for sparse reconstruction (GPSR) [11] *etc.* Besides the convex optimization methods, CS recovery can be implemented by some iterative greedy algorithms, e.g., orthogonal matching pursuit (OMP) [12].

### 2.2. Compressed Sensing with Non-Uniform Compressible Signals

The investigation of utilizing compressible signals' statistic characteristics has already attracted a lot of interest. The most influential one is the Bayesian compressive sensing (BCS) [13] which recovers the signal by estimating its posterior density function. With different signal models, there are several BCS schemes. The sparse Bayesian learning algorithm in [13] invokes a hierarchical prior to simplify the Laplace prior of compressible signal. The authors in [14] also use Laplace priors. The BCS via belief propagation is proposed in [15] by assuming that the compressible signal obeys the mixture Gaussian model. In [16] the authors combine the belief propagation and Bayesian learning algorithms together to provide a more universal and low-complexity BCS scheme.

However, these compressible signal probability density functions are still based on the assumption that the significant elements are uniformly distributed. Considering the image signals with non-uniform distribution, the wavelet tree model is introduced in BCS in [17]. Besides BCS, the prior information of compressible signal is also used in other ways. In [18], a new weighted rule for the iterative reweighted *l*_1_-norm minimization algorithm is integrated by explore the hidden Markov tree model of the wavelet coefficients. In [19], a weighted sampling scheme is proposed according to the statistics of image signals, where the weight matrix is determined by parameters of the direct-current and alternating-current components' distributions. The authors of [20] and [21] design unequal protection of the wavelet sub-bands in different scales by sampling with different random Gaussian matrices, and their difference is the measurement allocation strategy.

Compared with the unequal protection methods like the hidden Markov tree model in [18]. which uses an extra training process to estimate the exact statistics feature of the signal, the proposed EW-CS scheme firstly only need a coarse sparse support distribution trend as the prior knowledge, e.g., the well-known fact that the significant DCT or DWT coefficients appear at the low frequency. This makes the EW-CS scheme be widely used and easily implemented. From this point, the EW-CS is similar to the schemes presented in [20] and [21]. Nevertheless, the unequal protection mechanism of our scheme is quite different from theirs. They treat each sub-band (or parts with different importance) separately, but we provide nested windows to exploit the combined effect of different parts. With this design, the protection of the significant elements is further enhanced such that the overall performance of the EW-CS outperforms that of [21].

## 3. Proposed EW-CS Scheme

The proposed EW-CS scheme is detailed in this section. First, the non-uniform compressible signal is formularized. Then, the general EW-CS encoding and decoding algorithms are presented. Finally, we simplify the EW-CS to a two-window case which is quite important for applications and analysis.

### 3.1. Non-Uniform Compressible Signal

In the proposed EW-CS scheme, for clearer explanation, we assume that the signal *X* = {*x_i_* | *i* = 1,2,…*N*}*^T^* is compressible in the canonical basis, *i.e.*, the orthogonal basis Ψ*_N_*_×_*_N_* = *I*. The scheme can be directly applied to other basis. A more tractable definition of the compressible signal *X* is using the power law that the *k*-th largest entry of the signal obeys |*x_(k)_*| ≤ *C_p_*·*k*^−1/^*^p^*, where *C_p_* > 0 and 0 <p <1. *C_p_* represents the energy of the signal which is a constant depending on *p*, and *p* is the speed of decay. The sparsity is defined as *K* when there are *K* significant elements containing the most energy of the compressible signal, *i.e.*, the first *K* largest elements |*x_(k)_*|, *k* = 1,…*K*.

The non-uniform compressible signal means that the distribution of the significant elements in the sparse support is non-uniform, that is, the support distribution has some structured characteristics. We use importance classes to describe this distribution. From the sparsity perspective, the importance of a class is corresponding to how many significant elements it contains. The compressible signal *X* is divided into *W* non-overlapped importance classes. The size of the *w*-th class is defined by *s_w_* so that $N=\sum_{w=1}^{W}{\text{S}}_{w},$ *w* = 1, 2, …, *W*, 0 ≤ *s_w_* ≤ *N*. We further assume that the importance of a class decreases with the class's index, *i.e.*, the *w*-th class is more important than the (*w* + 1)-th class. The division can be described using a generating polynomial (GP) $\Pi =\sum_{w-1}^{W}\Pi_{w}x^{w}$, where the parameter Π*_k_* = (*s_k_/N*) ϵ [0,1] is the ratio of the elements in the importance class *w* to all the *N* elements. The sparsity distribution is given as $\Lambda (x)=\sum_{w-1}^{W}\Lambda_{w}x^{w},$ where 0 ≤ Λ*_w_* ≤ 1 is the proportional factor representing the number of significant elements placed in the *w*-th class.

### 3.2. General EW-CS

#### 3.2.1. Encoding

**Figure 2.**
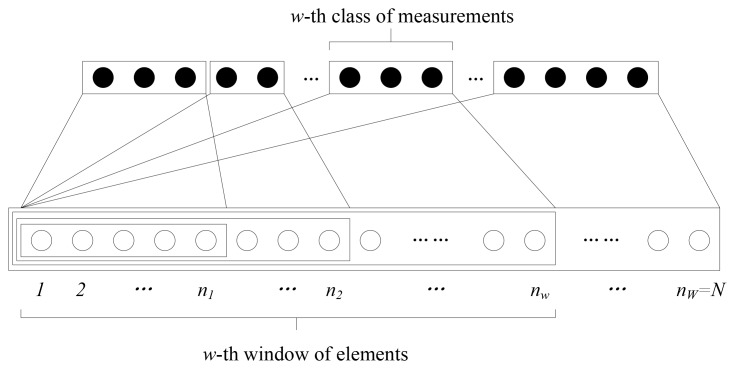
Expanding window compressed sensing.

The importance class determines a sequence of strictly increasing subsets of the signal's elements, which we call windows. The *w*-th window consists of all the first *w* classes, so the size of the window is, $n_{w}=\sum_{w-1}^{W}s_{k}$ *w* = 1, 2, …, *W*; thus, the last window contains all the signal's elements with *n_w_* = *N*. The structure of the EW-CS is shown in Figure 2.

In the classical CS algorithm, one method to generate the random sensing matrix is by picking the entries from Gaussian distribution *N* (0,1). Given the overall sensing rate *R*, the number of the random projections is *M* = *RN*. The measurements allocated for each window can also be given by a measuring polynomial (MP) $\Omega (x)=\sum_{w-1}^{W}\Omega_{w}x^{w},$ where 0 ≤ Ω*_w_* ≤ 1 denotes the ratio of measurements assigned for the *w*-th window to all the measurements. It means that we generate *m_w_* = Ω*_w_M* random projections (the *w*-th class of measurements as shown in Figure 2) for the *n_w_* elements of the *w*-th window, and $\sum_{w-1}^{W}m_{w}=M$. So the sensing matrix Ф*^w^* is a *m_w_* × *n_w_* random Gaussian matrix. The measurement vector of *w*-th window is calculated as $Yw=\Phi_{m_{w}\times n_{w}}^{w}X_{\left(w\right)},$ where *X_(w)_* is the vector of the elements in *w*-th window, and *X_(w)_* = *X*.

It can be noticed from Figure 2 that the *w*-th importance class belongs to the *w*-th window and all its subsequent windows, so the elements of *w*-th importance class are sampled by the *w*-th and all the subsequent classes of measurements. For example, the first importance class (also the first window) is sampled in all *M* measurements, and the last importance class is sampled only in Ω*_w_M* measurements. Therefore, the elements in importance class with smaller index corresponding to more measurements obtain more protection.

Although the classes of measurements are described to be generated sequentially in Figure 2, we can use a random selection strategy to introduce diversity. Each measurement is randomly assigned to a window according to a designed window selection distribution $\Gamma (x)=\sum_{w-1}^{W}\Gamma_{w}x^{w},$ where Γ*_w_* is the probability that the *w*-th window is chosen. Encoding with the window selection distribution may bring uncertain sensing rate, but it can be roughly regarded that *m_w_* = Γ*_w_M*. As in a CS system each measurement is actually the inner product of the corresponding row of the sensing matrix and the signal vector, it can be calculated separately. For instance, if one measurement $y_{j}^{\left(k\right)}$ *k* = 1, 2, …, *m_w_*, *j* = 1, 2, …, *M* is the *k*-th measurement assigned to the *w*-th window, we get $y_{j}^{\left(k\right)}={\left(\phi_{j}^{\left(k\right)}\right)}^{T}X(w),$ where $\phi_{j}^{\left(k\right)}$ is a random Gaussian vector with length *n_k_* and ${\left(\phi_{j}^{\left(k\right)}\right)}^{T}$ can be regarded as the *k*-th row of the sensing matrix $\Phi_{m_{w}\times n_{w}}^{w}$. This random window selection design may bring the robustness to the degradation caused by burst loss of measurements.

#### 3.2.2. Decoding

**Figure 3.**
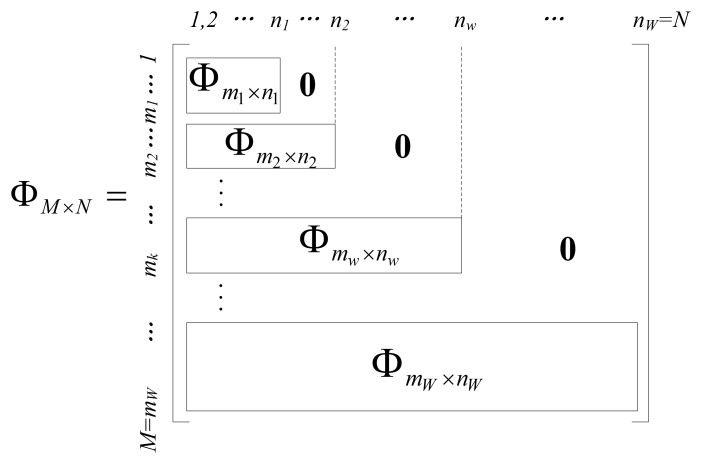
Joint sensing matrix for the general EW-CS.

The encoding strategy of the EW-CS is presented class by class. This is sometimes necessary for constrained encoder or distributed coding scenarios, but it is usually more efficient to use joint decoder, which combines the measurements together, and recovers the original *X* simultaneously through CS reconstruction method. If we use the sequential generation method, the joint sensing matrix Ф*_M_*_×_*_N_* can be written as a combination of all the window sensing matrix Ф*^w^*, as shown in Figure 3. The blank blocks are all zeros. Comparing with the ordinary CS sensing matrix, the constructed sensing matrix in Figure 3 is sparser so that the encoding computational complexity of our scheme is lower than that of ordinary CS. If we use the random window selection method, the sensing matrix is written as $\Phi_{M\times N}=\{\phi_{1}^{T};\phi_{2}^{T};\cdots ;\phi_{j}^{T};\cdots ;\phi_{M}^{T}\}$.

Thus, the joint decoding can be implemented using this joint sensing matrix. At the decoder side, all the received random projections compose a measurement vector $Y={\left\{y_{j}|j=1,2,\ldots ,M\right\}}^{T}={\left\{Y_{w}^{T}|w=1,2,\ldots ,W\right\}}^{T}$ which is generated by the sensing matrix Ф*_M_*_×_*_N_*, *i.e.*, *Y* = Ф*_M_*_×_*_N_X*. Thus, the signal *X* can be recovered using ordinary CS recovery algorithms mentioned in Section 2.

### 3.3. EW-CS with Two Windows

In the previous subsection, we described the proposed EW-CS scheme in general format. Nevertheless, the most common situation is that the significant elements of a natural compressible signal are concentrated at a certain part of the signal. Therefore, two importance classes are enough for the usual non-uniform compressible signal. The first importance class consists of more large elements than the second importance class. The first importance class is also the first window, and the entire signal is the second window. The two-window EW-CS has wider application prospects; thus, this special case of the EW-CS is discussed in detail below.

In the two-window EW-CS, the GP is Π(*x*) = Π_1_*x* + (1 − Π_1_)*x*^2^ which is determined by only one coefficient Π_1_ ∈ [0,1]. Similarly, the sparsity distribution can be written as Λ(*x*) = Λ_1_*x* + (1 − Λ_1_) *x*^2^. If the first class really has more significant elements, 0.5 < Λ_1_ ≤ 1; otherwise 0 < Λ_1_ ≤ 0.5. For clarification, we adopt the sequential generation as shown in Figure 2, so the MP is Ω(*x*) = Ω_1_*x* + (1−Ω_1_)*x*^2^. By increasing Ω_1_, we progressively increase the protection of the first importance class. The extreme cases of Π_1_ = M_1_ = 0 and Π_1_ = M_1_ = 0 are the ordinary CS problems.

Based on above definitions, the joint sensing matrix is given as:

(6) $$\Phi_{M\times \text{N}}=\left(\begin{array}{cc} \Phi_{1}^{m_{1}\times {\text{n}}_{1}} & 0\\ \Phi_{2,1}^{m_{2}\times n_{2}} & \Phi_{2,2}^{m_{2}\times (n_{2}-n_{1})} \end{array}\right),M=m_{1}+m_{2}\;\text{and}\;N=n_{2}$$

where $\Phi_{1}^{m_{1}\times n_{1}}$ is the sensing matrix for the first window (using Ф_1_ for short form), and $\Phi_{2}^{m_{2}\times n_{2}}=\left(\begin{array}{cc} \Phi_{2,1}^{m_{2}\times n_{1}} & \Phi_{2,2}^{m_{2}\times (n_{2}-n_{1})} \end{array}\right)$ is the sensing matrix for the second window (using Ф_2_ = (Ф_2,1_ Ф_2,2_) for short form). Then the encoding procedure can be given as:

(7) $$\begin{array}{ccc} Y_{1}=\Phi_{1}X_{\left(1\right)}, & Y_{2}=\Phi_{2}X_{\left(2\right)}=\Phi_{2}X, & Y={\left[\begin{array}{cc} Y_{1}^{T} & Y_{2}^{T} \end{array}\right]}^{T} \end{array}$$

where *Y*_1_ and *Y*_2_ denote the measurement vectors for the two windows respectively. The encoding can also be written in joint coding format that:

(8) $$Y=\left(\begin{array}{c} Y_{1}\\ Y_{2} \end{array}\right)=\left(\begin{array}{cc} \Phi_{1} & 0\\ \Phi_{2,1} & \Phi_{2,2} \end{array}\right)\;\left(\begin{array}{c} X_{1}\\ X_{2} \end{array}\right)=\Phi X$$

It must be noted that *X*_1_ and *X*_2_ denote the signal elements of the two importance classes not the two windows, which are defined as different from *X*_(1)_ and *X*_(2)_. The joint coding format is necessary for facilitating the performance analysis of the EW-CS and contrast with the ordinary CS in the next section.

## 4. Recovery Error Upper Bounds Analysis of the EW-CS

In this section, the reconstruction error upper bounds of the EW-CS is analyzed and contrasted with the ordinary CS scheme (using CS for short). The simple two-window EW-CS is firstly discussed, and then the results are further extended to the general EW-CS.

### 4.1. EW-CS with Two Windows

The *l*_2_-norm recovery error defined as ‖X − X̂‖*_l2_* is adopted to judge the recovery performance. The performance analysis is presented by contrasting between the CS and the EW-CS. According to the joint coding format of the EW-CS in Subsection 3.3, the CS problem is also formulated by correspondingly divided the signal vector *X*, the measurement vector *Y* and the sensing matrix Ф into different parts. It is given as:

(9) $$Y=\left(\begin{array}{c} Y_{1}^{\text{CS}}\\ Y_{2}^{\text{CS}} \end{array}\right)=\left(\begin{array}{cc} \Phi_{1,1} & \Phi_{1,2}\\ \Phi_{2,1} & \Phi_{2,2} \end{array}\right)\;\left(\begin{array}{c} X_{1}\\ X_{2} \end{array}\right)=\Phi^{\text{CS}}X$$

The most direct relationship between the CS and the EW-CS can be obtained by replacing the sub-matrix Ф_1,2_ with all zero matrix of the same size, and then the CS problem becomes the EW-CS as shown in Equation (8). In CS, the measurement vectors of two windows are calculated as:

(10) $$\begin{array}{c} Y_{1}^{\text{CS}}=\Phi_{1,1}X_{1}+\Phi_{1,2}X_{2}\\ Y_{2}^{\text{CS}}=\Phi_{2,1}X_{1}+\Phi_{2,2}X_{2} \end{array}$$

while in the EW-CS they are:

(11) $$\begin{array}{l} Y_{1}^{\text{EW}}=\Phi_{1,1}X_{1}\\ Y_{2}^{\text{EW}}=\Phi_{2,1}X_{1}+\Phi_{2,2}X_{2} \end{array}$$

It can be found that the only difference between the CS and the EW-CS is the different generation of the first class of measurements, *i.e.*, *Y*_1_*^CS^* and *Y*_1_*^EW^*. However, the joint recovery algorithms for the CS and the EW-CS use both of the two classes of measurements, so we need a joint coding representation to reveal the difference shown in Equations (10) and (11). Firstly, the compressible signal *X* can be written as $X=X_{1}^{\prime }=X_{2}^{\prime }$. The *N* dimensional vector $X_{1}^{\prime }$ is identical to the vector *X* for the first *n*_1_ elements, and the remaining (*n*_2_ − *n*_1_) elements of $X_{1}^{\prime }$ are set to zero. Similarly, the vector $X_{1}^{\prime }$ is identical to *X* for the last (*n*_2_ − *n*_1_) elements, and the remaining elements of $X_{2}^{\prime }$ are set to zero. Then, the encoding of CS is given as:

(12) $$Y^{\text{CS}}=\Phi^{\text{CS}}X=\Phi^{\text{CS}}\left(\left(\begin{array}{c} X_{1}\\ 0 \end{array}\right)+\left(\begin{array}{c} 0\\ X_{2} \end{array}\right)\right)=\Phi^{\text{CS}}\left(\begin{array}{c} X_{1}\\ 0 \end{array}\right)+\Phi^{\text{CS}}\left(\begin{array}{c} 0\\ X_{2} \end{array}\right)=\Phi^{\text{CS}}X_{1}^{\prime }+\Phi^{\text{CS}}X_{2}^{\prime }$$

while the encoding of the EW-CS is rewritten as:

(13) $$Y^{\text{EW}}=\Phi^{\text{EW}}X=\Phi^{\text{EW}}\left(\left(\begin{array}{c} X_{1}\\ 0 \end{array}\right)+\left(\begin{array}{c} 0\\ X_{2} \end{array}\right)\right)=\Phi^{\text{EW}}\left(\begin{array}{c} X_{1}\\ 0 \end{array}\right)+\Phi^{\text{EW}}\left(\begin{array}{c} 0\\ X_{2} \end{array}\right)=\Phi^{\text{EW}}X_{1}^{\prime }+\Phi^{\text{EW}}X_{2}^{\prime }$$

For analyzing the discriminational protection of different importance classes, we should consider *X*_1_ and *X*_2_ separately. This is equal to considering $X_{1}^{\prime }$ and $X_{2}^{\prime }$. If the joint recovery is regarded for recovering $X_{1}^{\prime }$ only, the Equations (12) and (13) are further rewritten as:

(14) $$Y^{\text{CS}}=\Phi^{\text{CS}}X_{1}^{\prime }+{\text{N}}_{e,1}^{\text{CS}},\text{where}\;N_{e,1}^{\text{CS}}=\Phi^{\text{CS}}X_{2}^{\prime }=\left(\begin{array}{c} \Phi_{1,2}X_{2}\\ \Phi_{2,2}X_{2} \end{array}\right)$$

(15) $$Y^{\text{EW}}=\Phi^{\text{EW}}X_{1}^{\prime }+{\text{N}}_{e,1}^{\text{EW}},\text{where}\;N_{e,1}^{\text{EW}}=\Phi^{\text{EW}}X_{2}^{\prime }=\left(\begin{array}{c} 0\\ \Phi_{2,2}X_{2} \end{array}\right)$$

The first terms on the right hand of Equations (14) and (15) are the same according to Equations (8) and (9), so the difference between the CS and the EW-CS is the noise on the $X_{1}^{\prime }$'s measurements. It is obvious that the energy of the noise in the CS scheme is larger than that in the EW-CS scheme. Intuitively, the recovery quality of $X_{1}^{\prime }$ in the EW-CS is better than in the CS.

This result can also be proved with the aid of mathematical formulae. From the Theorem 2 in [22], the *l*_2_-norm recovery error from inaccurate measurements have the upper bound as:

(16) $${\left\|\hat{f}-f\right\|}_{\ell_{2}}\leq C_{1,S}\cdot ɛ+C_{2,S}\cdot \frac{{\left\|f-f_{S}\right\|}_{\ell_{1}}}{\sqrt{S}}$$

where *f_s_* denotes the truncated vector corresponding to the *S* largest values of compressible signal *f* (in absolute value). *S* is chosen such that *δ*_3_*_S_* + 3*δ*_4_*_S_* < 2, and *f*'s sparsity *K* ≤ *S. ε* is the maximum *l*_2_-norm of the noise *e*, *i.e.*, ∥*e*∥_ℓ2_ ≤ *ε*. The constants *C*_1_,*_S_* and *C*_2_,*_S_* depend on the reasonable value of *δ*_4_*_S_*. The second term on the right hand side of Equation (16) is the approximation error in noiseless case which dominates the recovery error when the noise is small. It is obvious that this term becomes zero when the signal is exactly sparse. This upper bound is based on the assumption that the measurements are sufficient so that the signal is perfectly recovered.

For the $X_{1}^{\prime }$, all the measurements contribute to the recovery, so we can assume that the measurements are enough. Based on the result in Equation (16), the error upper bounds for the CS and the EW-CS respectively are given as:

(17) $${\left\|{\hat{X}}_{1}^{\prime \text{CS}}-X_{1}^{\prime }\right\|}_{\ell_{2}}\leq C_{1,S_{1}}\cdot {ɛ}_{1}^{\text{CS}}+C_{2,S_{1}}\cdot \frac{{\left\|X_{1}^{\prime }-X_{1,S_{1}}^{\prime }\right\|}_{\ell_{1}}}{\sqrt{S_{1}}}$$

(18) $${\left\|{\hat{X}}_{1}^{\prime \text{EW}}-X_{1}^{\prime }\right\|}_{\ell_{2}}\leq C_{1,S_{1}}\cdot {ɛ}_{1}^{\text{EW}}+C_{2,S_{1}}\cdot \frac{{\left\|X_{1}^{\prime }-X_{1,S_{1}}^{\prime }\right\|}_{\ell_{1}}}{\sqrt{S_{1}}}$$

Note that the second terms on the right side of Equations (17) and (18) are the same for the same $X_{1}^{\prime }$. The only difference of the recovery performance of $X_{1}^{\prime }$ between the CS and the EW-CS is on the noise error. According to Equations (14) and (15), the noise energy of the CS and the EW-CS has the relation as below:

(19) $${\left\|N_{e,1}^{\text{CS}}\right\|}_{\ell_{2}}^{2}={\left\|\Phi_{1,2}X_{2}\right\|}_{\ell_{2}}^{2}+{\left\|\Phi_{2,2}X_{2}\right\|}_{\ell_{2}}^{2}\geq {\left\|\Phi_{2,2}X_{2}\right\|}_{\ell_{2}}^{2}={\left\|N_{e,1}^{\text{EW}}\right\|}_{\ell_{2}}^{2}$$

and the equality holds when *X*_2_ is all zero vector. Based on Equation (19), it can be considered that *ε*_1_*^EW^* ≤ *ε*_1_*^CS^*. It means that the upper bound of the EW-CS for $X_{1}^{\prime }$ is lower than CS, and it reflects the higher recovery quality in all probability. Therefore, the more important part of signal *X*, *i.e.*, the subset of elements in $X_{1}^{\prime }$, is indeed better protected using the EW-CS. Next, if the joint recovery is regarded for $X_{2}^{\prime }$ only, the formulae (12) and (13) are rewritten as:

(20) $$Y^{\text{CS}}=\Phi^{\text{CS}}X_{2}^{\prime }+N_{e,2}^{\text{CS}}=\left(\begin{array}{c} \Phi_{1,2}X_{2}\\ \Phi_{2,2}X_{2} \end{array}\right)+N_{e,2}^{\text{CS}},\text{where}\;N_{e,2}^{\text{CS}}=\Phi^{\text{CS}}X_{1}^{\prime }=\left(\begin{array}{c} \Phi_{1,1}X_{1}\\ \Phi_{2,1}X_{1} \end{array}\right)$$

(21) $$Y^{\text{EW}}=\Phi^{\text{EW}}X_{2}^{\prime }+N_{e,2}^{\text{EW}}=\left(\begin{array}{c} 0\\ \Phi_{2,2}X_{2} \end{array}\right)+N_{e,2}^{\text{EW}},\text{where}\;N_{e,2}^{\text{EW}}=\Phi^{\text{EW}}X_{1}^{\prime }=\left(\begin{array}{c} \Phi_{1,1}X_{1}\\ \Phi_{2,1}X_{1} \end{array}\right)$$

Comparing with Equations (14) and (15), the information term and the noise term are exchanged, so in this case it is found that the noise for the CS and the EW-CS is the same, while the measurements of $X_{2}^{\prime }$ are different. It can be predicted from Equation (16) that the recovery error upper bounds for $X_{2}^{\prime }$ of the CS and the EW-CS vary on the approximation error term. However, the approximation error term in Equation (16) is based on the assumption that the measurements are enough for best recovery. The measurements for $X_{2}^{\prime }$ constitute only (1 − Ω_1_) percentage of all the measurements, so they may not guarantee the best recovery. Oher looser bounds for approximation error are introduced here for analyzing $X_{2}^{\prime }$. Of cause, the looser bounds can also be substituted into Equations (17) and (18) for analyzing $X_{1}^{\prime }$, and results are also the same as we've already given.

For compressible signals obeying the power law, there is an earlier result in [1] and [2] which states that the estimation from accurate measurements with minimal *l*_1_-norm obeys the following result with overwhelming probability:

(22) $${\left\|\hat{f}-f\right\|}_{\ell_{2}}\leq C_{p,\alpha }{\left(\frac{M}{\log N}\right)}^{-\left(\frac{1}{p}-\frac{1}{2}\right)}$$

where *M* ≥ log*N* is the number of measurements, and *N* is the length of signal *f*. $C_{p,\alpha }=C_{P,\alpha }^{\prime }\cdot C_{p}$ is a constant depending only on the signal energy *C_p_*, the fixed decay speed *p* and *α*. The parameter *α* is a sufficiently small positive number to ensure Equation (22) is behaved with high probability. The bound in Equation (22) also holds for the compressible signal with ∥*f*∥_ℓ1_*R* and *p* = 1 when $C_{p,\alpha }=C_{P,\alpha }^{\prime }\cdot R$. By substituting Equation (22) into Equation (16), we get the upper bound as:

(23) $${\left\|\hat{f}-f\right\|}_{\ell_{2}}\leq C_{1,S}\cdot ɛ+C_{p,\alpha }{\left(\frac{M}{\log N}\right)}^{-\left(\frac{1}{p}-\frac{1}{2}\right)}$$

This is a looser one than Equation (16), for there is a probability of failure although it is quite trivial. If we assume that the component signal $X_{2}^{\prime }$ is still a compressible signal obeying the power law, the recovery error upper bounds of $X_{2}^{\prime }$ for the CS and the EW-CS then can be given as:

(24) $${\left\|{\hat{X}}_{2}^{\prime CS}-X_{2}^{\prime }\right\|}_{\ell_{2}}\leq C_{1,S_{2}}\cdot {ɛ}_{2}^{\text{CS}}+C_{p2,\alpha }{\left(\frac{M_{2}^{\text{CS}}}{\log N}\right)}^{-\left(\frac{1}{p_{2}}-\frac{1}{2}\right)}$$

(25) $${\left\|{\hat{X}}_{2}^{\prime \text{EW}}-X_{2}^{\prime }\right\|}_{\ell_{2}}\leq C_{1,S_{2}}\cdot {ɛ}_{2}^{\text{EW}}+C_{p2,\alpha }{\left(\frac{M_{2}^{\text{EW}}}{\log N}\right)}^{-\left(\frac{1}{p^{2}}-\frac{1}{2}\right)}$$

where *M*_2_ is the number of effectual measurements for $X_{2}^{\prime }$. From Equations (20) and (21) we know that the noise error terms of Equations (24) and (25) are the same, *i.e.*, ${ɛ}_{2}^{\text{EW}}={ɛ}_{2}^{\text{CS}}={ɛ}_{2}$; nevertheless, $M_{2}^{\text{CS}}=M$ for ordinary CS, and $M_{2}^{\text{EW}}=(1-\Omega_{1})\cdot M$, where Ω_1_ is defined in previous section as the ratio of number of measurements assigned to the first window (*i.e.*, the more important part *X*_1_). Obviously, $M_{2}^{\text{EW}}<M_{2}^{\text{CS}}$, so weighing the recovery quality of $X_{2}^{\prime }$ in the CS and the EW-CS cases against each other depends on the value of *p*_2_. It is easy to find that when 0 < *p*_2_ < 2, the CS's bound is lower than the EW-CS's; when *p*_2_ = 2, they are equal; when *p*_2_ > 2, the EW-CS's bound is lower than CS's in return. However, the error bound is behaved for compressible signal usually with 0 < *p*_2_ < 1 or with 0 < *p*_2_ < 2 [2] in the broadest sense. So the aforementioned comparison leads to the result that the recovery quality of $X_{2}^{\prime }$ in the EW-CS is worse than that in CS with high probability.

Now, the comparison between the CS and the EW-CS for the entire signal *X* is discussed. The square error of signal *X* is given as:

(26) $${\left\|\hat{X}-X\right\|}_{\ell_{2}}^{2}={\left\|{\hat{X}}_{1}-X_{1}\right\|}_{\ell_{2}}^{2}+{\left\|{\hat{X}}_{2}-X_{2}\right\|}_{\ell_{2}}^{2}={\left\|{\hat{X}}_{1}^{\prime }-X_{1}^{\prime }\right\|}_{\ell_{2}}^{2}+{\left\|{\hat{X}}_{2}^{\prime }-X_{2}^{\prime }\right\|}_{\ell_{2}}^{2}-{\left\|\hat{X}\right\|}_{\ell_{2}}^{2}$$

Thus, the overall recovery error upper bounds for *X* using the CS and the EW-CS can be derived by substituting Equations (17), (18), (24) and (25) into Equation (26) such that:

(27) $${\left\|{\hat{X}}^{\text{CS}}-X\right\|}_{\ell_{2}}^{2}\leq \left[C_{1,S_{1}}^{2}\cdot {\left({ɛ}_{1}^{\text{CS}}\right)}^{2}+A\cdot {ɛ}_{1}^{\text{CS}}\right]+\left[B{\left(M_{2}^{\text{CS}}\right)}^{-(\frac{2}{p_{2}}-1)}+D{\left(M_{2}^{\text{CS}}\right)}^{-(\frac{1}{p_{2}}-\frac{1}{2})}\right]+E-{\left\|{\hat{X}}^{\text{CS}}\right\|}_{\ell_{2}}^{2}$$

(28) $${\left\|{\hat{X}}^{\text{EW}}-X\right\|}_{\ell_{2}}^{2}\leq \left[C_{1,S_{1}}^{2}\cdot {\left({ɛ}_{1}^{\text{EW}}\right)}^{2}+A\cdot {ɛ}_{1}^{\text{EW}}\right]+\left[B{\left(M_{2}^{\text{EW}}\right)}^{-(\frac{2}{p_{2}}-1)}+D{\left(M_{2}^{\text{EW}}\right)}^{-(\frac{1}{p_{2}}-\frac{1}{2})}\right]+E-{\left\|{\hat{X}}^{\text{EW}}\right\|}_{\ell_{2}}^{2}$$

where $A=2C_{1,S_{1}}C_{2,S_{1}}\frac{{\left\|X_{1}^{\prime }-X_{1,S_{1}}^{\prime }\right\|}_{\ell_{2}}}{\sqrt{S_{1}}}$, $B=C_{p,\alpha }^{2}{\left(\log N\right)}^{\left(\frac{2}{p_{2}}-1\right)}$, $D=2C_{1,S_{2}}C_{p,\alpha }{\left(\log N\right)}^{\left(\frac{1}{p_{2}}-\frac{1}{2}\right)}{ɛ}_{2}$, $E=C_{2,S_{1}}^{2}\cdot \frac{{\left\|X_{1}^{\prime }-X_{1,S_{1}}^{\prime }\right\|}_{\ell_{2}}}{S_{1}}+C_{1,S_{2}}^{2}\cdot {\left({ɛ}_{2}\right)}^{2}$.

We assume that ${\left\|{\hat{X}}^{\text{CS}}\right\|}_{\ell_{2}}^{2}\approx {\left\|{\hat{X}}^{\text{EW}}\right\|}_{\ell_{2}}^{2}$. From the analysis above, we've known that the first term in right hand side of Equation (28) is smaller than Equation (27) but the second term is larger, so the comparison between Equation (27) and Equation (28) is undetermined. With the same settings, the difference between the upper bounds of the CS and the EW-CS is a function depending only on *ε*_1_ and *M*_2_ which are determined by sparsity distribution and MP, *i.e.*, the parameters Λ_1_ and Ω_1_. However, if there are really more significant elements placed in $X_{1}^{\prime }$, *i.e.*, 0.5 < Λ_1_ ≤ 1, the first term will dominate the overall error upper bounds; otherwise, the second term will do so. Thus, the EW-CS is superior to the CS for a non-uniform compressible signal with proper importance classes assignment.

### 4.2. General EW-CS

Based on the ana1ysis of the two-window EW-CS, the results are easily extended to *W* ≥ 3 windows case. The encoding of the *W*-window EW-CS is written as:

(29) $$\begin{array}{c} Y^{\text{EW}}=\left(\begin{array}{ccccc} \Phi_{1,1} & \cdots & 0 & \cdots & 0\\ \vdots & \ddots & \vdots & 0 & \vdots \\ \Phi_{1,w} & \cdots & \Phi_{w,w} & \cdots & 0\\ \vdots & ⋰ & \vdots & \ddots & \vdots \\ \Phi_{W,1} & \cdots & \Phi_{W,w} & \cdots & \Phi_{W,w} \end{array}\right)\;\left(\begin{array}{c} X_{1}\\ \vdots \\ X_{w}\\ \vdots \\ X_{W} \end{array}\right)\\ =\Phi^{\text{EW}}\left(\left(\begin{array}{c} X_{1}\\ \vdots \\ 0\\ \vdots \\ 0 \end{array}\right)+\cdots +\left(\begin{array}{c} 0\\ \vdots \\ X_{w}\\ \vdots \\ 0 \end{array}\right)+\cdots +\left(\begin{array}{c} 0\\ \vdots \\ 0\\ \vdots \\ X_{w} \end{array}\right)\right)=\Phi^{\text{EW}}(X_{1}^{\prime }+\cdots +X_{w}^{\prime }+\cdots +X_{W}^{\prime }) \end{array}$$

Note that the EW-CS's sensing matrix Φ*^EW^* is a lower triangular matrix. We only consider one importance class $X_{w}^{\prime }\in {\mathbb{R}}^{N}$ at a time, and assume that it is compressible obeying the power law; thus, the looser bound in Equation (23) is used. So the recovery error upper bound of the elements in this class is:

(30) $${\left\|{\hat{X}}_{w}^{\prime CS}-X_{w}^{\prime }\right\|}_{\ell_{2}}\leq C_{1,S_{w}}\cdot {ɛ}_{w}^{\text{CS}}+C_{p_{w},\alpha }{\left(\frac{M_{w}^{\text{CS}}}{\log N}\right)}^{-\left(\frac{1}{p_{w}}-\frac{1}{2}\right)}$$

(31) $${\left\|{\hat{X}}_{w}^{\prime \text{EW}}-X_{w}^{\prime }\right\|}_{\ell_{2}}\leq C_{1,S_{w}}\cdot {ɛ}_{w}^{\text{EW}}+C_{p_{w},\alpha }{\left(\frac{M_{w}^{\text{EW}}}{\log N}\right)}^{-\left(\frac{1}{p_{w}}-\frac{1}{2}\right)}$$

Comparing with the conventional CS, only for the first importance class vector $X_{1}^{\prime }$, the second terms on the right hand sides of Equations (30) and (31) are the same, so the first class is always better protected in the EW-CS than in the CS. For the last importance class vector $X_{W}^{\prime }$, the first terms are the same, and the second term of the EW-CS has high probability to be larger than the CS; thus, the last importance class is usually sacrificed. For other importance classes, the two terms on the right hand side are both different from the CS, so it is hard to judge the protection level comparing with the CS. However, it can be instinctively known that the smaller *w* is, the *w*-th importance class has better recovery quality. Finally, the overall error upper bound for the EW-CS is given as:

(32) $${\left\|{\hat{X}}^{\text{EW}}-X\right\|}_{\ell_{2}}^{2}=\sum_{w=1}^{W}{\left\|{\hat{X}}_{w}^{\text{EW}}-X_{w}\right\|}_{\ell_{2}}^{2}=\sum_{w=1}^{W}{\left\|{\hat{X}}_{w}^{\prime \text{EW}}-X_{w}^{\prime }\right\|}_{\ell_{2}}^{2}-(W-1)+{\left\|{\hat{X}}^{\text{EW}}\right\|}_{\ell_{2}}^{2}$$

## 5. Variations of the EW-CS

**Figure 4.**
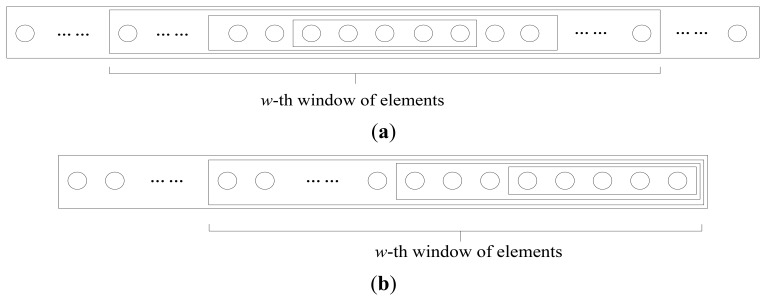
The variations of the expanding windows. (**a**) The most important class is in the middle. (**b**) The most important class is at the back.

During the introduction of the EW-CS scheme in Section 3, there may be a question that what happens if the importance of classes doesn't decrease with the class index as we assumed. From the analysis in last section, we know that the performance of the EW-CS degraded badly when the more important class doesn't contain more significant elements, *i.e.*, 0.5 < Λ_1_ ≤ 1 in the two-window case. Thus, the extension of the EW-CS is necessary for this case. The two basic variations are shown in Figure 4 that the window design can changed according to the location of the important class. If the significant elements concentrate in several parts of the signal, the same class of window can also be divided into several parts to match. Nevertheless, the benefit of using the EW-CS in this case will be not worth the complexity it causes; thus, it may be better to use other methods or the CS.

It doesn't mean that the EW-CS can only be used in the simple cases where the significant elements concentrate in the front, in the middle or at the back of the signal. It has an even more complicated variation in a certain case. Recall that we have designed a window selection distribution such that each measurement can select the window it belongs to. Similarly, the signal element can also choose the window that it belongs to. It looks a little abnormal in our usual practice, for the signal element may not know its own importance or even do the selection. However, it can be realized in certain case, the wireless sensor network scenarios.

Let us consider the CWS [4] problem. The *N* sensors' data is regarded as a compressible signal X = {*x_i_* | *i* = 1, 2, …, *N*}, where *x_i_* is the data of the *i*-th sensor. Using ordinary CS, each sensor send *y_ij_* = *φ_ji_x_i_* to the sink node, and then the sink can collect all the received data to generate one measurement $y_{j}=\sum_{i-1}^{N}\varphi_{ij}x_{i}$. Repeat this process for several times until the sink get enough measurements to recover the compressible signal *X*. This mechanism assumes that all the sensors' data are equal importance, but it is a common situation in wireless sensor networks that a warning line is set *a priori* for the temperature, pressure or gas strength detection systems. In this case, the EW-CS scheme can be used to simplify this collection process. Each sensor can decide its sensed data is important or not according to the warning line, and then choose the window it belongs to. With one warning line, the two-window EW-CS is applied. If the sensor's data is beyond the warning line, it is in the first window and sends its *y_ji_* for *M* times. If its data is within the security region, it is in the second window and sends its *y_ji_* for only *m*_2_ < *M* times. This leads to the better recovery quality for the warning sensor's data, and resaves the transmission energy for the safe sensors.

## 6. Experimental Results

### 6.1. Simulation Results for Synthetical Signals

In our simulation, the *l*_2_-norm of the recovery error, *i.e.*, ∥ *X* − *X̂* ∥_ℓ2_, is used to evaluate the performance of the proposed EW-CS algorithm. A compressible signal *X* ∈ ℝ*^N^* is generated obeying the power law with the parameters *C_p_* = 10 and *p* = 1 The length of the signal is *N* = 512, and the sparsity is set as *K* = 50. The two-window EW-CS is used to compare with the ordinary CS. For constructing the signal in a non-uniform format, the randomly selected Λ_1_*K* elements from the first *K K* largest elements are placed in the first importance class, and the rest (1−Λ_1_)*K* elements are placed in the second importance class. Other (*N*−*K*) smaller elements are distributed randomly. The parameters defining the size of the first importance class and its corresponding number of measurements are fixed as Π_1_ = 0.3 and Ω_1_ = 0.5.

The overall sensing rate is *R*, and the total number of measurements is *M*. The sensing matrix Ф*^EW^* is constructed by first creating a *M* × *N* matrix, *i.e.*, the sensing matrix Ф*^CS^*, with i.i.d. draws of a Gaussian distribution *N*(0,1), then the right upper Ω_1_*M* × (1−Π_1_)*N* sub-matrix is replaced by all zero matrix with the same size. The recovery algorithm we used is the *l*_1_ based method which is implemented by using a package for specifying and solving convex programs [23,24].

**Figure 5.**
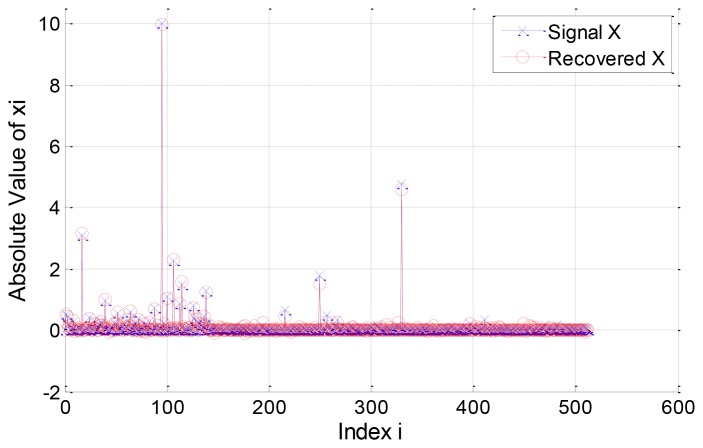
The non-uniform compressible signal *X* and its reconstruction using the EW-CS.

In the first example, we consider a compressible signal with Λ_1_ = 0.8 and *R* = 0.4. The original signal and its recovered version are plotted in Figure 5. The *l*_2_-norm error for the component signals $X_{1}^{\prime }$ and $X_{1}^{\prime }$ are 4.9488 and 11.2609 respectively, and the *l*_2_-norm error for the signal *X* is 1.1808. Note that the significant elements of the signal are almost well recovered and the elements in the first importance class have better recovery quality than that in the second class.

**Figure 6.**
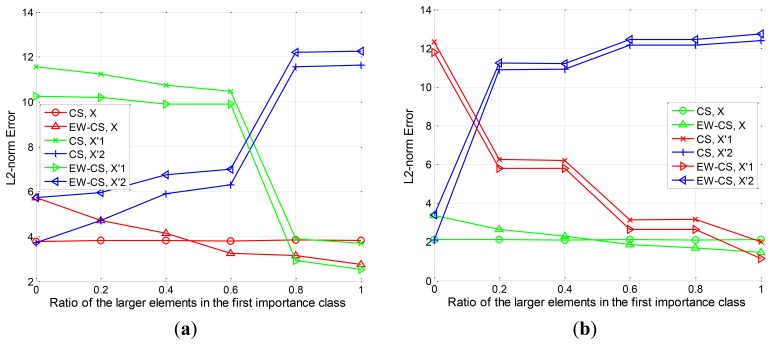
The *l*_2_-norm recovery error with different Λ_1_. (**a**) *R* = 0.2, (**b**) *R* = 0.4.

For various choices of Λ_1_, the *l*_2_-norm error of the signal *X* and the component signals $X_{1}^{\prime }$ and $X_{2}^{\prime }$ using the proposed EW-CS and ordinary CS is shown in Figure 6. We ran each value 100 times, and simulations are presented for overall sensing rates *R* = 0.2 and *R* = 0.4. The results indicate that the performance of the EW-CS varies with the ratio Λ_1_, while the ordinary CS is not impacted by Λ_1_. With the increase of Λ_1_, the recovery error of the EW-CS decreases. Roughly speaking, when 0 ≤ Λ_1_ ≤ 0.5, the CS outperforms the EW-CS. This because that the first importance class is not the really most important one. It means that using the EW-CS with wrong matched window settings will introduce more errors.

Nevertheless, when 0.5 ≤ Λ_1_ ≤ 1, the EW-CS begins to outperform the CS. In this case, the window settings match the signal's features, so the superiority of the EW-CS is demonstrated. It can also be noticed that the performance for the component signal $X_{1}^{\prime }$ in the EW-CS is always superior to that in the CS. It verifies the theoretical result proved in Subsection 4.1 that the elements in the first class are better protected. However, the performance for the component signal $X_{2}^{\prime }$ in the EW-CS is worse than that in the CS. This also agrees with our analysis.

### 6.2. Simulation Results for Natural Images

For testing the practical performance of the proposed EW-CS, instead of using artificially generated signals, we used real-world non-uniform compressible signals. The image signals' DCT or DWT coefficients constitute non-uniform compressible signals. With this prior knowledge, the random sensing with the EW-CS can be accomplished. The 512 × 512 test gray images with different features, *i.e.*, “Lena”, “Boat”, “Peppers” and “Aerial”, are used for simulation. The images are divided into 32 × 32 blocks for computational facility.

The M-CS scheme in [21] is simulated here for comparison. M-CS needs to do 2-D DWT at the encoder for exploiting the frequency bands feature of wavelet coefficients. Then they used the *l*_1_ based method to recover the wavelet coefficients, and did the inverse wavelet transform to recover the original image. In our simulation, the three level DWT is used in M-CS with the measurement allocation rule in [21]. The level-3 (the lowest band) is assigned 60 measurements, and the number of measurements of level-1 is approximately four times of level-2.

Although the importance class design in the EW-CS can also directly use the sub-bands of 2-D DWT coefficients, the additional transform process is not willing to do. Thus, we choose 1-D DCT as the sparse representation basis which will be used only at the decoder so that the low-complexity encoder is guaranteed. The ordinary CS is also simulated with the 1-D DCT.

**Figure 7.**
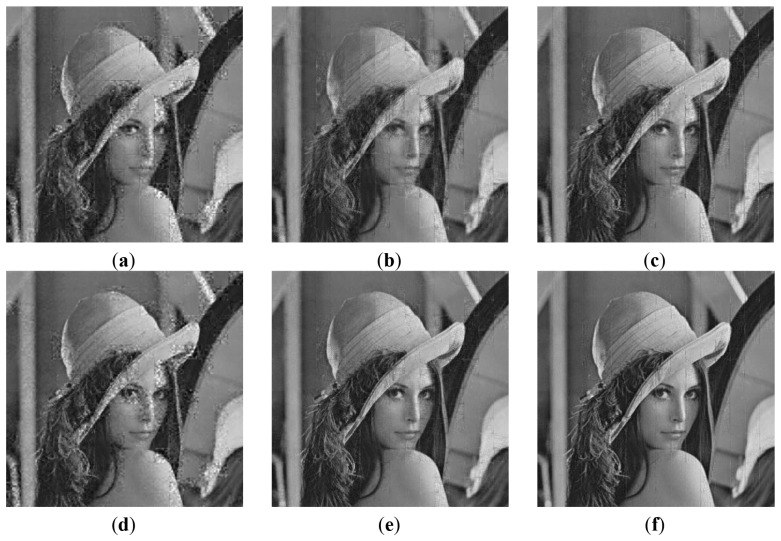
Visual quality comparisons at sensing rate *R* = 0.2 and *R* = 0.4 for 512 × 512 “Lena” using the proposed EW-CS, the M-CS [21] and the CS. (**a**) M-CS, *R* = 0.2, (**b**) Ordinary CS, *R* = 0.2, (**c**) EW-CS, *R* = 0.2, (**d**) M-CS, *R* = 0.4, (**e**) Ordinary CS, *R* = 0.4, (**f**) EW-CS, *R* = 0.4.

**Figure 8.**
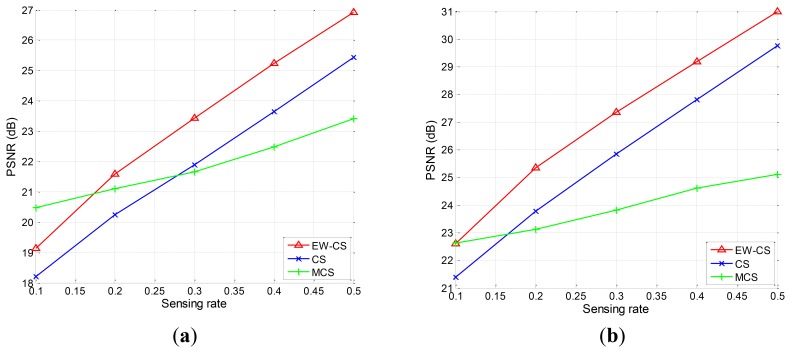

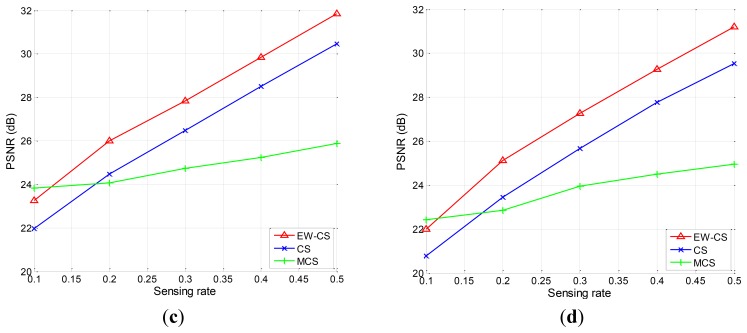
The PSNR performances with different sensing rates. (**a**) “Aerial”, (**b**) “Boat”, (**c**) “Lena”, (**d**) “Peppers”.

Firstly, we use the two-window EW-CS with the parameters Π_1_ = 0.3 and Ω_1_ = 0.5. The visual quality comparison for “Lena” is given in Figure 7 and the PSNR performances with different sensing rates of the four images are further shown in Figure 8. It is obvious that the proposed EW-CS scheme outperforms the other methods expect for a small number of low sensing rates. The significant coefficients of image signal really appear in the first window of the EW-CS such that they are well recovered. Thus, the EW-CS achieves better overall performance than the ordinary CS.

In M-CS, the significant coefficients are only sampled by the measurements allocated to the sub-band they belong, not all the measurements, so its performance is worse than the EW-CS at most sensing rates. At low sensing rate, the number of measurements for the most significant coefficients in the M-CS is fixed to guarantee successful recovery, and these measurements are noiseless. However, the EW-CS cannot recover the elements in the first importance class from the noisy measurements even though they are more. When the sensing rate increases, this problem will not exist.

**Figure 9.**
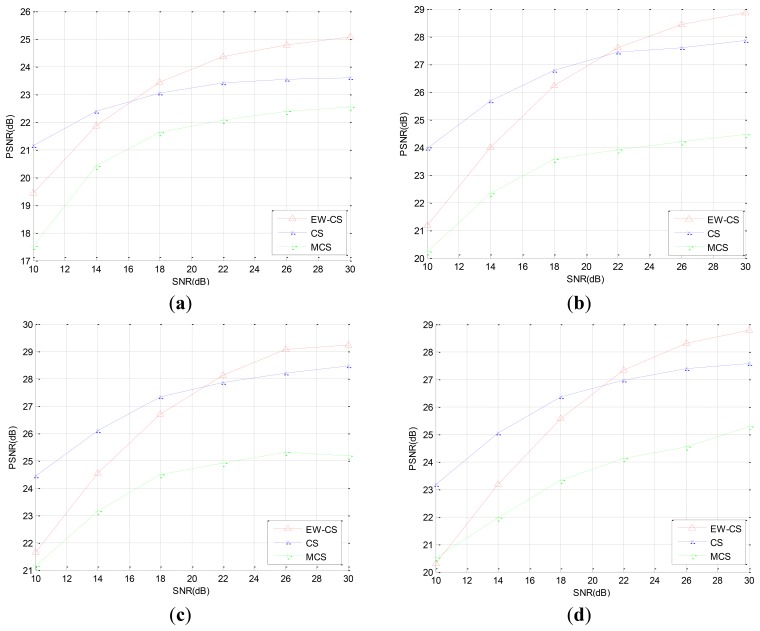
The PSNR performances with different SNR (dB). (**a**) “Aerial”, (**b**) “Boat”, (**c**) “Lena”, (**d**) “Peppers”.

In the second simulation, we reconstruct the images from the noisy measurements which are transmitted through an AWGN channel. The sensing rate is fixed as *R* = 0.4. In order to compare the PSNR performance of the original CS method and the EW-CS method, we fix the signal power, and compensate the power of the all zero matrix Φ_1,2_ to the Φ_1,1_ to enlarge the signal power of the most significant class. The PSNR performances with different signal-to-noise ratio (SNR) for the four images are shown in Figure 9.

We can observe that the EW-CS is worse than the CS at lower SNR, but outperforms the CS when the SNR increases. The M-CS's PSNR is lowest at the same SNR, but its decay speed is similar to the CS and is slower than the EW-CS. When the channel noise energy gets greater, the advantage of recovery the first importance class of the EW-CS is weaken, and the recovery quality of the second importance class degrades further. Thus, the EW-CS algorithm is more applicable to the mild transmission environments.

**Figure 10.**
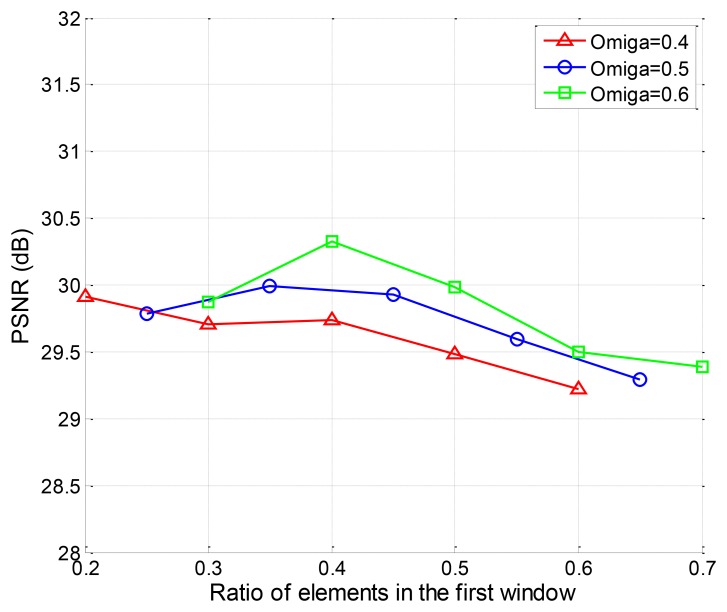
The PSNR performance with different first window size.

Then, we investigate the impact of changing window size on the recovery quality of the two-window EW-CS. The PSNR performance of “Lena” with different Π_1_ is given in Figure 10. The window design must follow a basic rule that the rank of the joint sensing matrix of the EW-CS must be larger than the total number of measurements, or the convex programming problem is an unsolved one. So the ranges of Π_1_ for different measurement allocation parameters Ω_1_ will be different. That's why the x axis for each curve in Figure 10 is different. For a fixed Ω_1_, there is a optimal first window size, but the general trend is the first window should not be too large.

**Figure 11.**
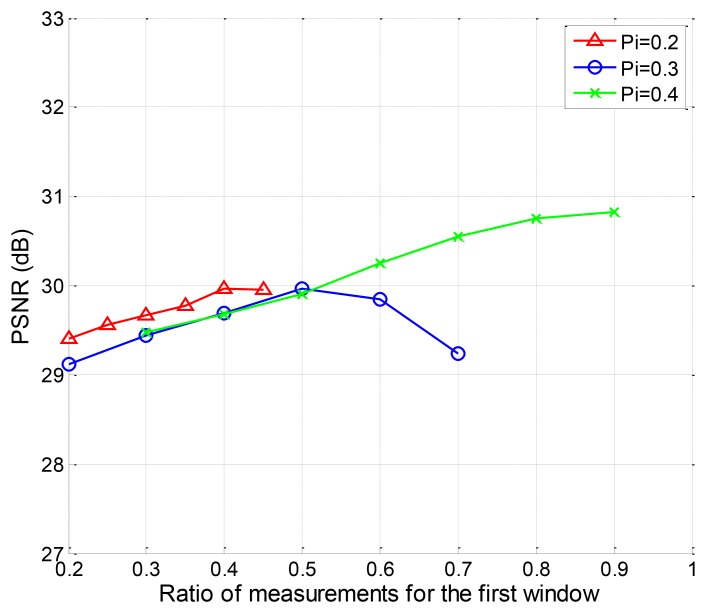
The PSNR performance with different number of measurements for the first window.

We also evaluate the PSNR performance of the EW-CS with different Ω_1_ when Π_1_ = 0.2, Π_1_ = 0.3 and Π_1_ = 0.4. The available ranges of Ω_1_ for different Π_1_ are different according to the design rule of the joint sensing matrix. The simulation results are plotted in Figure 11. It can be found that for the fixed first window size with Π_1_ = 0.2 and Π_1_ = 0.3, there is an optimal number of measurements that should be assigned to it. For Π_1_ = 0.4, however, the more measurements, the better.

**Figure 12.**
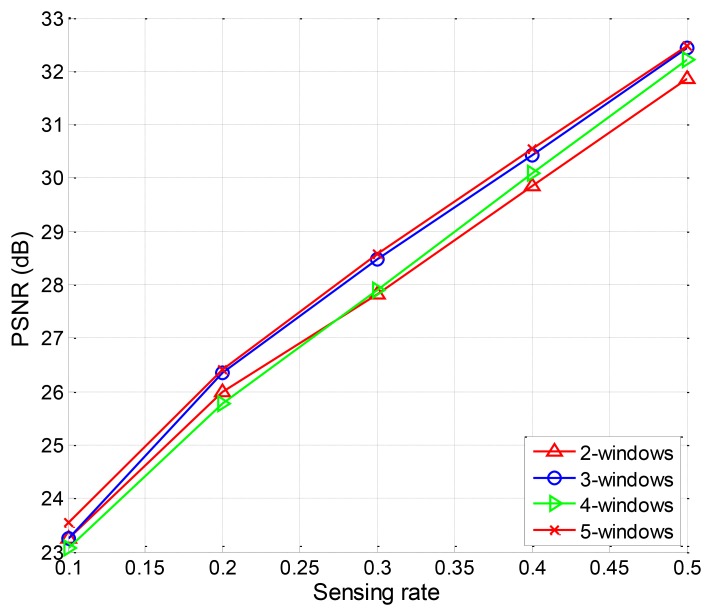
The PSNR performances for different number of windows.

Finally, we design the EW-CS with more than two windows. The rank law should also be obeyed. So the three-window EW-CS is designed with the GP Π(*x*) = 0.3*x* + 0.2*x*^2^ + 0.5*x*^3^ and MP Ω(x) = 0.5*x* + 0.2*x*^2^ + 0.3*x*. The four-window EW-CS is designed withΠ(*x*) = 0.1*x* + 0.2*x*^2^ + 0.3*x*^3^ + 0.4*x*^4^ and Ω(x) = 0.1*x* + 0.2*x*^2^ + 0.3*x*^3^ + 0.4*x*^4^. The five-window EW-CS is designed with Π(*x*) = 0.1*x* + 0.1*x*^2^ + 0.1*x*^3^ + 0.2*x*^4^ + 0.5*x*^5^ and Ω(*x*) = 0.1*x* + 0.1*x*^2^ + 0.3*x*^3^ + 0.2*x*^4^ + 0.3*x*^5^. The PSNR performance for different window designs for image “Lena” is shown in Figure 12. The differences among the four different window designs are trivial. It means that the simple EW-CS with two windows is competent in most image signal compression applications.

### 6.3. Simulation Results for Networked Data

The variation of the EW-CS applied in wireless sensor networks is implemented here. We define a 500 m × 500 m sensing area. *N* = 256 sensors are randomly distributed within this area to monitor the events of interest. When *E* events happen, the sensor's data is affected by all the events, and the relationship between them is assumed to be a distance decaying model, which is determined by the distance *d_i,j_* from the sensor *i* to the event *j*, where *i* = 1, 2, …, *N* and *j* = 1, 2, …, *E*, and the attenuation factor. If the *j*-th event's magnitude is *C_j_*, the *i*-th sensor's data is calculated as $S_{i}=\sum_{j-1}^{E}C_{j}d_{ij}^{-\alpha }$, where *α* is the propagation loss factor. Here we assume that *E* = 3, and all *C_j_* are equal to 100, *α* = 3.8.

**Figure 13.**
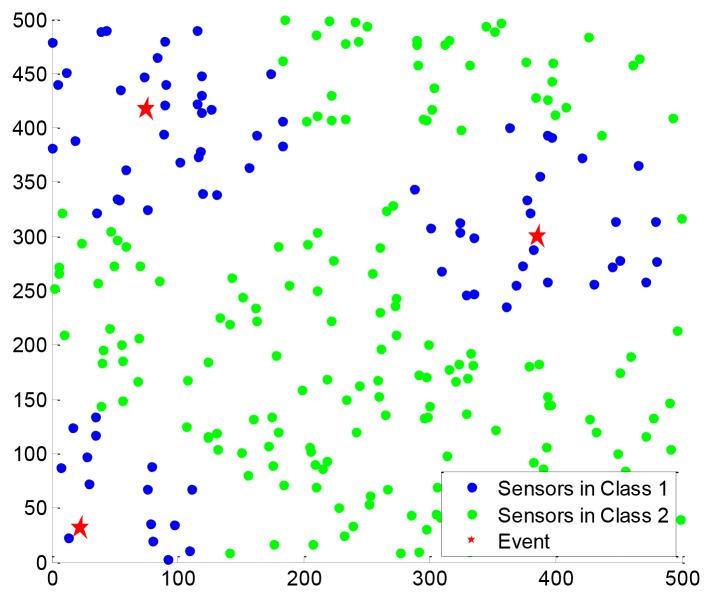
The random sensor network with three events (denoted by red stars) and two windows. The sensors in the first window are denoted by blue circles, and the other sensors are denoted by green nodes.

**Figure 14.**
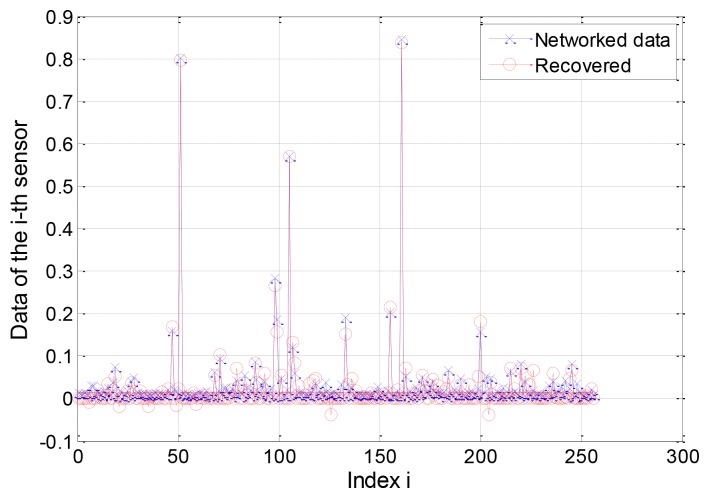
The networked data and its recovery signal.

**Figure 15.**
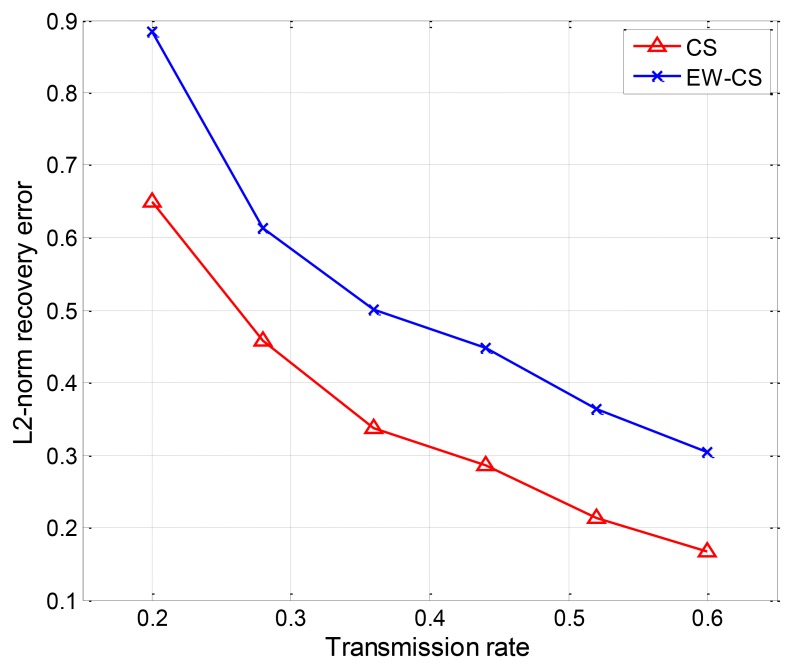
The *l*_2_-norm recovery error with different transmission rate.

**Figure 16.**
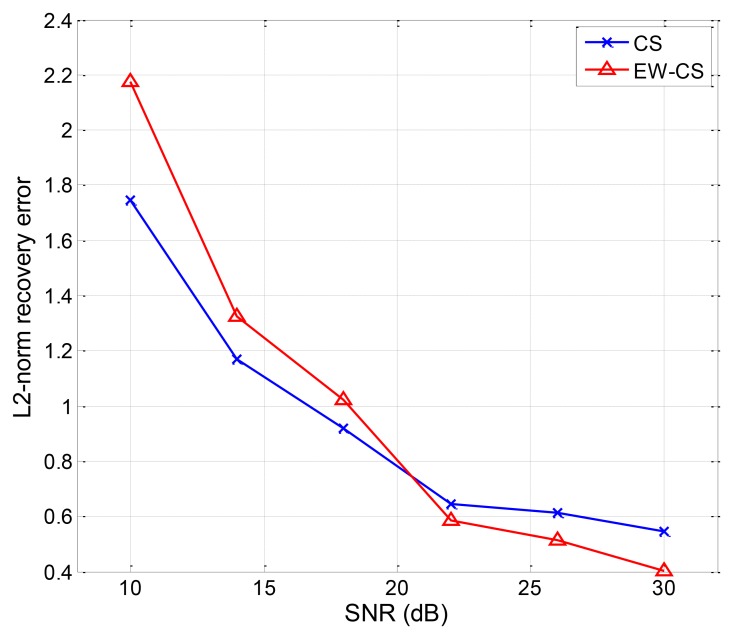
The *l*_2_-norm recovery error with different SNR.

For the two-window EW-CS, Figure 13 shows a scenario under our settings with the first window ratio Π_1_ = 0.3. The first window size is determined by the threshold given to each sensor from the collection centre (not shown in Figure 13). All the sensors transmit their data to the centre using one hop. As the EW-CS using less number of transmissions than the ordinary CS with the same sensing rate, we define the transmission rate (the ratio between the total number of transmissions and the number of sensors) as the criterion. The networked data in Figure 13 and its recovered version with the transmission rate 0.4 are shown in Figure 14, where Ω_1_ = 0.5. The *l*_2_-norm recovery error of this example is 0.2401. More simulation results with different transmission rates are plotted in Figure 15. The performance of the EW-CS is quite superior to the ordinary CS. Considering the transmission noise through the AWGN channel, the *l*_2_-norm recovery error with different SNR is given in Figure 16, where the transmission rate is fixed as 0.4. In the noisy case, the EW-CS performs similar as in image compression that it holds its advantage only at high SNR.

**Figure 17.**
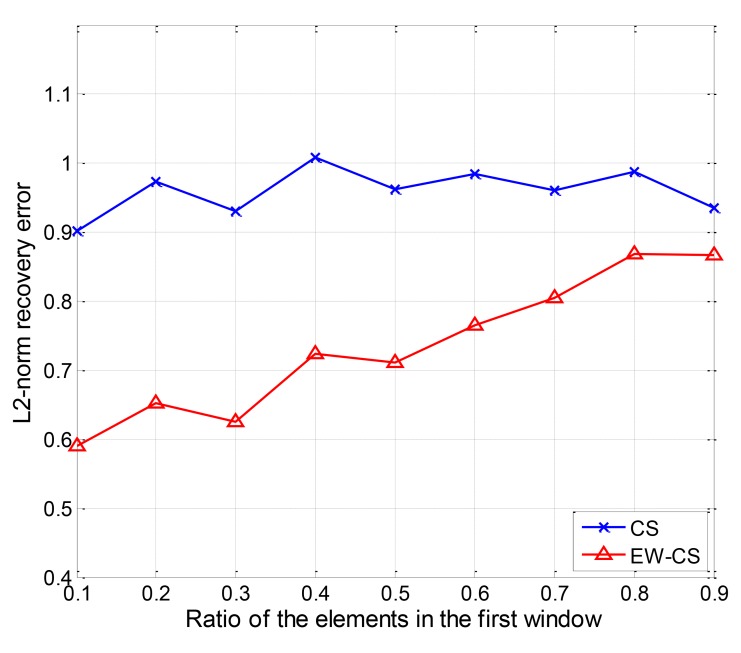
The *l*_2_-norm recovery error with size of the first window.

Finally, the performance of the EW-CS with changing first window size is shown in Figure 17, where Ω_1_ = 0.5 and the transmission channel is noiseless. The EW-CS in CWS case has the feature that the Π_1_*N* elements in the first window are expected to be the first Π_1_*N* largest ones. With the same ratio Ω_1_, the smaller the first window is, the more measurements are assigned to the most largest or significant elements, so the overall performance is inversely proportional to the size of the first window as shown in Figure 17. When the first window size approaches the entire network data, the EW-CS's performance gets closer to the ordinary CS.

## 7. Conclusions

A novel compressed sensing scheme called expanding window compressed sensing is proposed in this paper to provide unequal protection for non-uniform compressible signals. The efficiency of the proposed scheme is analyzed from the recovery error upper bounds perspective by comparing with ordinary compressed sensing. Different from weighted methods, the windowing technology is adopted to make the scheme more flexible and efficient. Comparing with the blocked sensing method, the nested window design gets more benefit from the joint recovery algorithm for the nested window. The scheme is further applied to practical non-uniform compressible signals, *i.e.*, image signals and networked data, to verify its superior performance. However, its noise-resilient performance is not very good, which is the problem we decided to solve in future work. The adaptive version of the proposed scheme that can optimize the parameters of designed windows is also an interesting direction to be investigated.
